# Novel endophytic fungus *Leptosphaeria* sp. strain T-2 improves plant growth and environmental stress tolerance

**DOI:** 10.1007/s44154-024-00186-6

**Published:** 2024-12-09

**Authors:** Taku Yamaguchi, Ryota Kataoka

**Affiliations:** Faculty of Life and Environmental Sciences, University of Yamanashi, Kofu, Yamanashi 400-0085 Japan

**Keywords:** Endophytic fungi, Drought and salinity stress, Plant tolerance, Drylands

## Abstract

Drought and salinity stress pose threats to agricultural production in drylands. Although breeding and genetic modification techniques have been employed to develop drought- and salt-tolerant crops, these methods are costly and risky. Hence, the potential application of endophytic fungi in dryland agriculture is being explored as a novel approach in improving plant tolerance to environmental stress. In this study, endophytic fungi with growth-promoting effects were isolated, characterized, and evaluated in terms of their ability to confer drought and stress tolerance to their host plants. Seventy-seven growth-promoting endophytic fungi belonging to 20 genera were isolated from barley roots; of these, strain T-2 elicited remarkable effects on plant growth parameters. Phylogenetic analysis revealed that strain T-2 belongs to genus *Leptosphaeria*, whose members are generally known as plant pathogens. Thus, *Leptosphaeria* sp. strain T-2 is a novel endophytic fungus that promotes plant growth. Moreover, it alleviated growth inhibition caused drought and salinity stress, as evidenced by the survival and maintained health of lettuce plants inoculated with strain T-2. The results of this study suggest that strain T-2 can be applied as a biofertilizer to improve agricultural production in drylands.

## Highlights

• Endophytic fungi with growth-promoting effects were isolated and identified.

• Phylogenetic analysis revealed fungal strain T-2 belongs to genus Leptosphaeria.

• Fungal strain T-2 improved plant growth under drought and salt stress.

• Fungal strain T-2 conferred drought and salt stress tolerance to its host plant.

• Fungal strain T-2 may be utilized in dryland agriculture.

**Graphical Abstract:**

## Graphical abstract

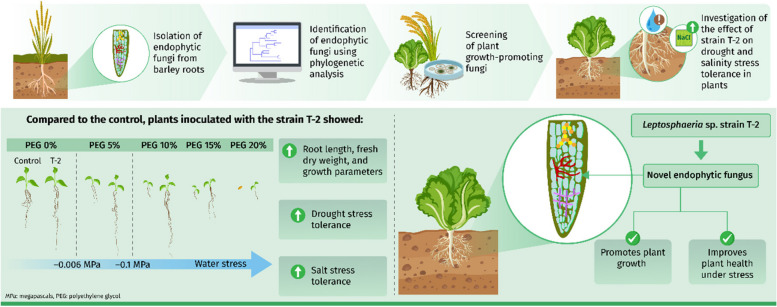

## Introduction

Drylands are one of the most important regions in the world (Huang et al. 2017): drylands constitute 41% of the total land area and are occupied by 38% of the world’s population; contain 45% of the world’s agricultural land (Burrell et al. 2020); and account for approximately 40% of the world’s net primary production. However, land degradation due to climate change and anthropogenic activities contributes to low soil fertility, leading to declining agricultural productivity in arid and semi-arid regions (Scheffer et al. 2001; Fu and An 2002; Rietkerk et al. 2004; Maestre et al. 2013; Li et al. 2016; Zhou et al. 2016).

As water is an important resource for plants, drought stress, mainly due to water deficiency, causes cellular dehydration and detrimentally affects essential plant processes (Hsiao 1973). Salinity stress also adversely affects plant growth. Soils with high salinity are common in arid regions; currently, approximately 20% of irrigated land is affected by salinity stress (Flowers et al. 1997). To overcome the negative effects of water and salinity stress on the morphology, physiology, biochemistry, and overall productivity of crops, drought-resistant xerophytes are being grown in drylands. Moreover, research aimed at enhancing the tolerance of plants to environmental stress through breeding and genetic modification has been conducted (Sallam et al. 2019; Kumar et al. 2020). However, such techniques have some disadvantages, including gene loss, high costs, and regulatory issue (Tsatsakis et al. 2017). For instance, the expansion of irrigation facilities requires securing sufficient water sources and large investments (Turral et al., 2010); moreover, breeding and genetic modification techniques necessitate the development of new individual varietie (Ahanger et al. 2017). To address these issues, the potential agricultural applications of endophytic fungi that coexist with plants are being investigated to develop a new approach in improving agricultural production in drylands.

Endophytic microorganisms are nonpathogenic bacteria and fungi that reside in plants. In natural ecosystems, a symbiotic relationship exists between plants and endophytic microorganisms (Zhao et al. 2010). Endophytic microorganisms form a complex ecological community that influences plant growth and productivity through their metabolic activities (Vurukonda et al. 2016). In particular, endophytic fungi provide water and nutrients to their host, thereby promoting plant growt (Rodriguez et al. 2009). Previous studies have demonstrated that endophytic fungi contribute to the water-absorption ability, environmental stress tolerance, and pest and disease resistance of their host plant (Bonfante and Genre 2010; Baum et al. 2015; Gill et al. 2016). Although not all plant–endophytic fungi relationships are mutualistic, some types of endophytic fungi positively affect plants by promoting their growth and protecting them against environmental stress (Arnold and Engelbrecht 2007; Kleczewski et al. 2012). These endophytic fungi could be used in dryland agriculture. For example, the endophytic fungus *Piriformospora indica*, discovered in plants in the desert regions of India in 1998, improves the growth and environmental stress tolerance of its wide range of hosts (Verma et al. 1998; Waller et al. 2005). It has been reported that it contributes to increased barley yield by promoting the growth and enhancing the resistance of barley to salt stress and disease. Other endophytic fungi such as *Penicillium* spp. and *Phoma glomerata* can significantly increase plant height under salinity and drought stress; specifically, *Phoma glomerata* significantly increased the height of inoculated plants under normal growth condition (Waqas et al. 2012). *Trichoderma* spp., a major group of rhizosphere microorganisms, promote plant growth and development (Harman et al. 2004; Qi and Zhao 2013). Some endophytic fungi are biological control agents: they suppress plant diseases caused by fungi, bacteria, nematodes, and insect (Ferreira et al., 2021). Recently, there are few reports on endophytic fungi that can tolerate water and salinity stress; these fungi can be used for promoting plant growth and sustainable agricultural production in drylands. However, such endophytic fungi have not been comprehensively isolated and identified.

Hence, this study was conducted to identify endophytic fungi that confer water and salinity stress tolerance to host plants. The findings of this study contribute to the development and implementation of strategies aimed at improving the water and salinity stress tolerance of crops in drylands.

## Results

### Isolation and phylogenetic analysis of endophytic fungi

Seventy-seven strains of endophytic fungi were isolated from the barley roots. These strains were grouped based on their morphological characteristics and the results of PCR–RFLP analysis. Based on the sequence analysis results of each group, at least 20 strains were identified at the genus level: *Talaromyces*, *Fusarium*, *Cephalosporium*, *Leptosphaeria*, *Humicola*, *Penicillium*, *Pyrenophora*, *Taifanglania*, *Alternaria*, *Ascomycota*, *Chaetomium*, *Chrysosporium*, *Geomyces*, *Microdochium*, *Mortierella*, *Pleosporales*, *Pseudogymnoascus*, *Purpureocillium*, *Setophoma*, and *Westerdykella*. The strain T-2 obtained in this study grew 11 days after the initiation of root-cut culture and was the only strain isolated from the same plant species. This strain was also isolated from plants collected from other fields in the same region, but was not isolated from the plants from other regions.

### PGP effect of strain T-2 on barley and lettuce

**Fig. 1:**
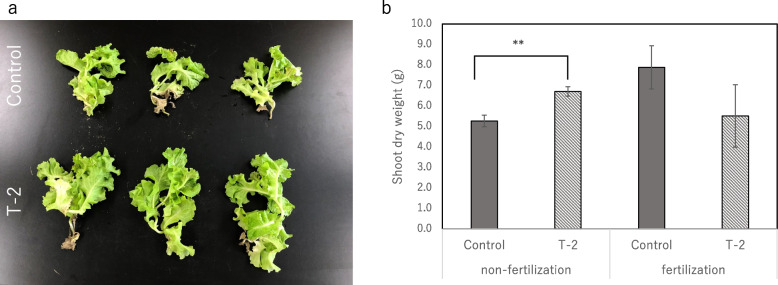
Effect of endophytic fungus strain T-2 inoculation on plant growth for lettuce cultivation under field conditions. Values are the means of three replicates (SE, *n* = 3), asterisks indicate significance corresponding to *P* < 0.01 (Student *t*-test)

**Fig. 2:**
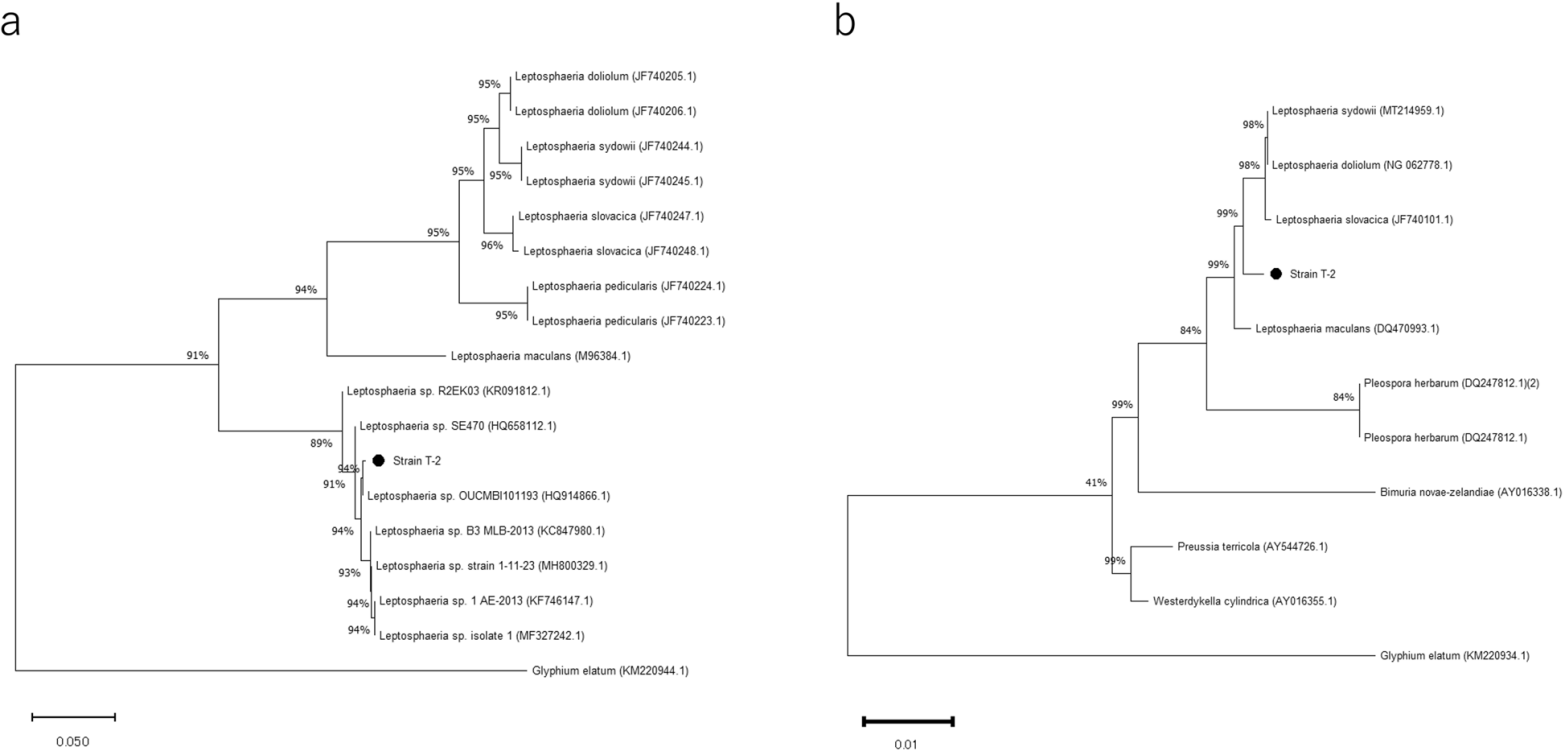
Phylogenetic tree of *Leptosphaeria* sp. T-2 isolated from barley roots based on the ITS region (**a**) and SSU rDNA sequences (**b**). The tree was generated using neighbor-joining analysis. Bootstrap values ​​(expressed as percentages of 1000 replicates) greater than 40% are indicated at branch points. Glyphium elatum is used as an outgroup

The growth parameters of the barley plants inoculated with strain T-2 significantly increased (*P* < 0.05): the ear FW increased by 41.3%, ear DW by 55.1%, shoot DW by 63.1%, and root DW by 41.4% (Table 1). Similar PGP effects were also observed in lettuce; the shoot FW and DW of the lettuce plants increased by 23.8% and 2.3%, respectively (Table 2). Strain T-2 promoted lettuce growth even after 30 days of field cultivation, as evidenced by a 27.3% increase in shoot and root DW (Fig. 1). Among the fungal strains isolated, strain T-2 showed the highest concordance with *Leptosphaeria* species (Accession number; LC813232), the phylogenetic analysis of its ITS region revealed that it belongs to genus *Leptosphaeria* (Fig. 2).

**Table 1:** Effects of *Leptosphaeria* sp. strain T-2 association on the barley growth in a growth chamber for 28 days

	Shoot length (cm)	Root length (cm)	Shoot fresh weight (g)	Shoot dry weight (mg)	Root fresh weight (g)	Root dry weight (mg)	Spike fresh weight (mg)	Spike dry weight (mg)
Control	41.2 ± 4.3	50.3 ± 4.1	2.5 ± 0.6	4.0 ± 1.3	1.3 ± 0.4	1.2 ± 0.3	3.7 ± 0.4	0.9 ± 0.2
T-2	46.2 ± 3.3	51.0 ± 13.7	3.3 ± 0.3	6.4 ± 0.5 **	1.1 ± 0.2	1.7 ± 0.2 *	5.2 ± 0.7 *	1.4 ± 0.3 *
Y1-25	41.8 ± 4.8	56.1 ± 23.9	2.0 ± 0.6	3.5 ± 1.3	0.6 ± 0.07 *	1.2 ± 0.4	3.0 ± 1.0	0.8 ± 0.3
Y2-19	41.9 ± 6.6	51.7 ± 20.8	2.0 ± 0.5	3.0 ± 1.0	0.6 ± 0.2 **	0.8 ± 0.3	2.8 ± 0.7	0.7 ± 0.3
G-4	42.6 ± 4.6	51.4 ± 9.2	2.8 ± 0.7	5.0 ± 1.8	0.8 ± 0.2 *	1.1 ± 0.3	3.9 ± 1.2	1.1 ± 0.4

Values are the means of three replicates (SE, *n* = 3), One or two asterisks indicate significance corresponding to *P* < 0.01 or *P* < 0.05, respectively (Student-*t* test)

**Table 2:** Effects of *Leptosphaeria* sp. T-2 strain inoculation on the vegetative growth of lettuce plants

	Shoot length (cm)	Shoot fresh weight (g)	Shoot dry weight (mg)
Control	16.7 ± 0.7	9.3 ± 0.7	641.7 ± 83.4
T-2	17.36 ± 0.6	12.3 ± 1.1**	656.4 ± 147.0

Values are the means of five replicates (SE, *n* = 5), asterisks indicate significance corresponding to *P* < 0.01 (Student-*t* test)

### Strain T-2 colonized the root tissue of barley plants

**Fig. 3:**
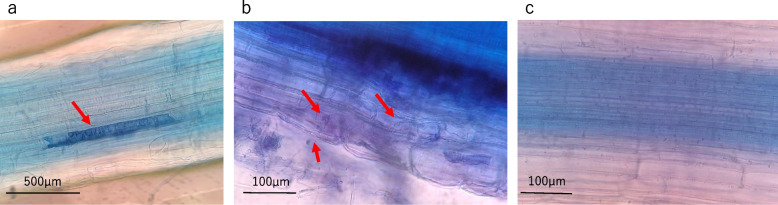
Colonization of *Leptosphaeria* sp. T-2 in barley roots. **a**, **b** Colonization of strain T-2 in the inoculated barley root. **c** Fungal colonization was not observed in the roots of barley grown under sterile conditions

**Fig. 4:**
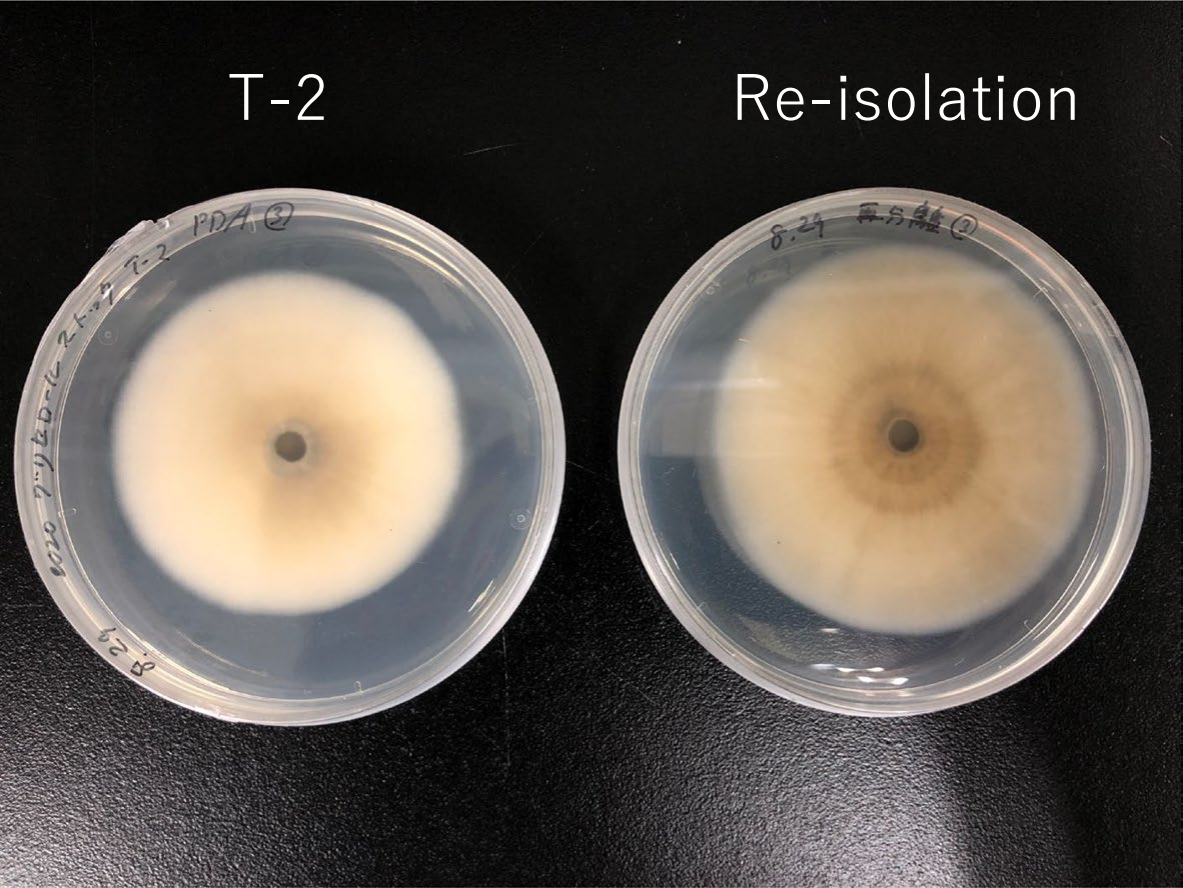
Re-isolation of strain T-2 from the roots of inoculated lettuce seedlings after 2 weeks

In the histological observation, hyphae were found in the intracellular and intercellular spaces in the root tissue of barley inoculated with the strain T-2 (Fig. 3a, b), whereas none were found in the root tissue of the sterile plants (Fig. 3c). In addition, the same endophytic fungus was reisolated from lettuce roots inoculated with the strain T-2 as evidence that the strain T-2 was established in the root tissue. The colony of strain T-2 has a characteristic: on PDA medium, the mycelium spreads in concentric circles, and the colony is milky white to gray at first, but gradually turns black from the center of the colony over time. As shown in Fig. 4, colonies of strains isolated from lettuce roots inoculated with strain T-2 and cultured for 2 weeks showed the same changes in colony morphology and pigmentation as those of strain T-2. The two colonies were highly homologous, with the reisolated strain showing 99% homology to strain T-2.

### Strain T-2 conferred drought and salt stress tolerance to its host plant (lettuce)

**Fig. 5:**
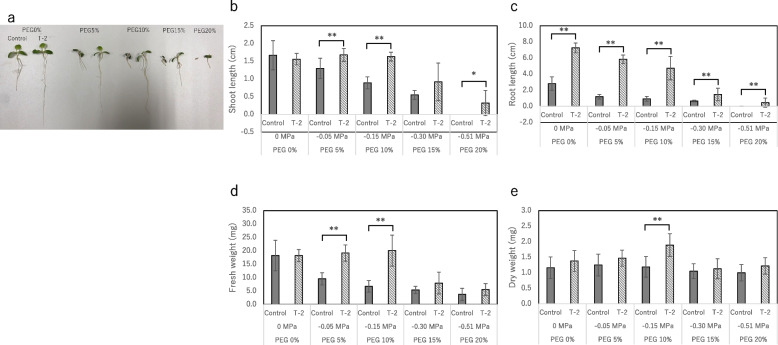
Effect of strain T-2 association and drought stress treatment on the plant growth. **a** Lettuce inoculated with strain T-2 cultivated under PEG concentration 0%-20%. **b**-**e** Lettuces were grown in a growth chamber under water stress for 7 days. In PEG 10%, one seed that did not germinate under strain T-2 inoculation conditions was excluded from the calculation. In PEG 20%, no seeds germinated in the control and five seeds did not germinate under strain T-2 inoculation conditions. The growth parameters of these seeds, shoot length and root length, were set to 0 cm, and the fresh weight and dry weight were calculated in the same way as the other parts. Values are the means of five replicates (SE, *n* = 10), One or two asterisks indicate significance corresponding to *P* < 0.01 or *P* < 0.05, respectively (Student *t*-test)

**Fig. 6:**
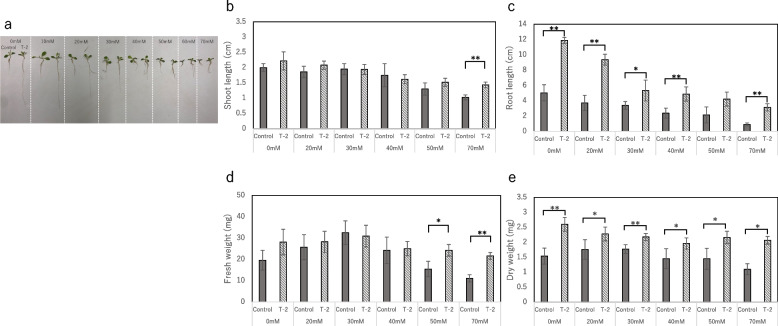
Effect of strain T-2 association and salt stress treatment on the plant growth. **a **Lettuce inoculated with strain T-2 cultivated under salt concentration 0 mM-70 mM. **b**-**e **Lettuces were cultivated in a growth chamber under salt stress for 10 days. In this experiment, seeds that did not germinate due to stress were excluded from the calculation. At 30 mM and 40 mM NaCl, one seed did not germinate in the control group. At 50 mM NaCl, five seeds did not germinate in the control group. At 60 mM NaCl, two did not germinate in the control group and one did not germinate in the strain T-2 inoculation condition. At 70 mM NaCl, one did not germinate in the control and strain T-2 inoculation conditions, two did not germinate in the control group, and two did not germinate in the strain T-2 inoculation condition. Values are the means of five replicates (SE, *n* = 5), One or two asterisks indicate significance corresponding to *P* < 0.01 or *P* < 0.05, respectively (Student *t*-test)

Although all growth parameters decreased under drought stress, the lettuce plants inoculated with strain T-2 remained healthy (Fig. 5), indicating that strain T-2 improves plant growth and alleviates drought stress. Similarly, the growth parameters of the lettuce plants gradually decreased under salinity stress, indicating that the plants remained healthy (Fig. 6). The growth parameters of the lettuce plants under salt stress (70 mM soil salinity) decreased compared with those of the plants grown under normal conditions (0 mM soil salinity). Nevertheless, the growth parameters of the lettuce plants inoculated with strain T-2 were higher than those of the control plants: SL decreased by 48.50% in the control and by 35.59% in the inoculated plants; RL decreased by 82.14% in the control and by 73.65% in the T-2–inoculated plants; FW decreased by 45.00% in the control and by 21.43% in the T-2–inoculated plants; and DW decreased by 26.67% in the control and by 19.23% in the T-2–inoculated plants. These results revealed that strain T-2 improved the salt stress tolerance of the lettuce plants and alleviated growth inhibition caused by salt stress.

**Fig. 7:**
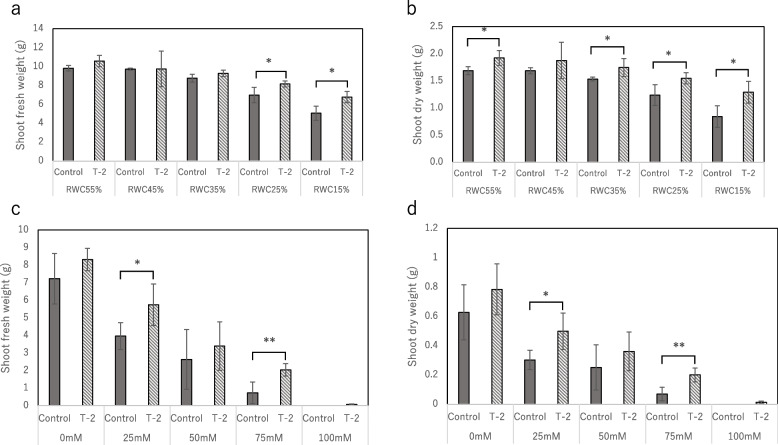
Effect of strain T-2 association on environmental stress tolerance in lettuce plants. **a** and **b **Lettuce was grown under water stress (RWC 55%-15%) for 28 days. **c** and **d** Lettuce was grown salinity stress (NaCl concentration 0 mM-100 mM) for 28 days. Values are the means of five replicates (SE, *n* = 5), One or two asterisks indicate significance corresponding to *P* < 0.01 or *P* < 0.05, respectively (Student *t*-test)

The effect of strain T-2 on improving plant tolerance to environmental stress was also observed in the 4-week pot culture; even under severe environmental stress, the health of the plants inoculated with strain T-2 improved. Figure 7a shows that compared with the plants grown under normal conditions (RWC 55%), the FW of the control and lettuce plants inoculated with strain T-2 under drought stress decreased. As a general trend, the decrease in FW at 15–25% RWCs was lesser in the plants inoculated with strain T-2 than in the control plants. A similar trend was observed in the DW of the control and lettuce plants inoculated with strain T-2 under drought stress. At different RWCs, the decrease in DW was lesser in the plants inoculated with strain T-2 than in the control plants (Fig. 7b). Therefore, strain T-2 inoculation alleviated growth suppression caused by drought stress. Figure 7c shows that compared with the plants grown under normal conditions (0 mM NaCl), the FW of the control and lettuce plants inoculated with strain T-2 under salinity stress decreased. As a general trend, the decrease in FW at different NaCl concentrations was lesser in the plants inoculated with strain T-2 than in the control plants. The dry weight was also compared in the same way as above: at NaCl 25 mM, 51.9% for the control and 36.4% for Strain T-2 inoculation; at NaCl 50 mM, 60.0% for the control and 54.0% for Strain T-2 inoculation; at NaCl 75 mM, 89.0% for the control and 74.5% for strain T-2 inoculation; at NaCl 100 mM, all control plants were dead and T-2 plants were 98.3% dead (Fig. 7d). These results revealed that under salinity stress, inoculation with strain T-2 alleviated plant growth inhibition. These results indicate that the negative effects of drought and salinity stress on plant health were ameliorated by inoculation with strain T-2, even during long-term cultivation.

## Discussion

In this study, fungal strain T-2, which promotes plant growth, was isolated from barley roots. The phylogenetic analysis of its ITS and SSU regions revealed that it belongs to genus *Leptosphaeria* (Spatafora et al. 2006; Piątek et al. 2020; Kodsueb et al. 2006). The PGP effect of strain T-2 was noticeable without fertilization; however, no significant effects were observed when fertilization occurred. Thus, the PGP effect of strain T-2 is only manifested in oligotrophic soil. Several studies have been conducted on the microbial materials produced by endophytic fungi; however, the effects observed in stable environments, such as growth chambers, become hardly noticeable when plants are cultivated in complex environments. The uncertainty of obtaining the beneficial effects of endophytic fungi on crops cultivated in a complex outdoor environment limits the potential application of endophytic fungi as a microbial material in agriculture. In addition, as some endophytic fungi elicit positive effects on the survival of host plants, the feasibility of using endophytic fungi as biofertilizers has been investigated. Bacteria and fungi can be utilized as biofertilizers because they increase nutrient availability in soil through their biological activities. Some endophytic fungi form mutualistic relationship with several tree specie (Knapp et al. 2012). Endophytic fungi are known to aid plant growth and development, as roots colonized by endophytic fungi can absorb and store nitrogen, phosphorus, and iron for longer periods than non-colonized roots (Mohamed et al. 2022). *Penicillium pinophyllum* is an endogenous biofertilizer that improves pomegranate production in semi-arid areas and helps plants take up potassium and phosphat (Wahid and Mehana 2000; Maity et al. 2019). *Piriformospora indica* is a biofertilizer with various properties that promotes plant growth and increases plant stress tolerance (Pal et al. 2015). Hence, fungal strain T-2 can be used as a biofertilizer because infected seedlings can be easily cultivated in advance in commercially available Jiffy-7® peat pellets; moreover, it can be used as a microbial material that is effective under a wide range of environmental conditions.

**Fig. 8:**
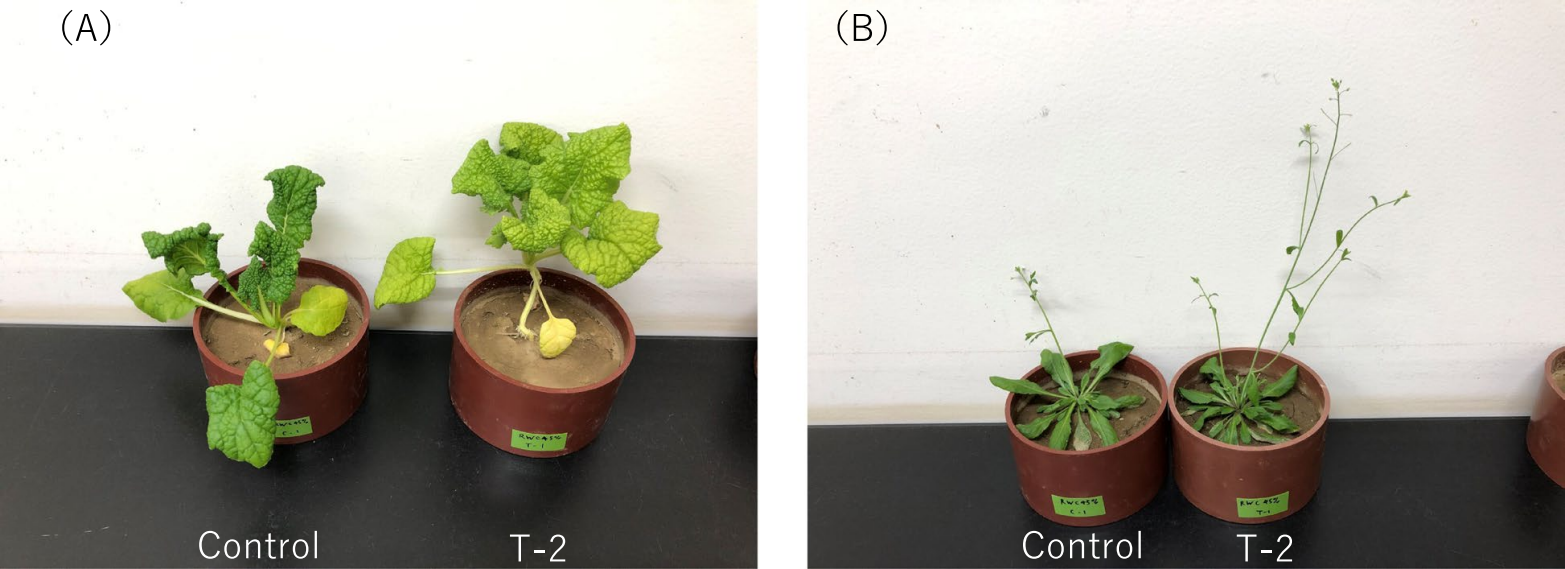
Plant pathogenicity of *Leptosphaeria* sp. strain T-2. **A** oilseed rape and **B** *Arabidopsis thaliana*, which have been associated with strain T-2

Although *Leptosphaeria maculans* is a well-known pathogen that causes *Brassica* root rot disease (Sprague et al. 2009), there are few reports that it is a PGP fungus. As shown in Fig. 1, there were no major differences between strain T-2 and *Leptosphaeria maculans*. In addition, when strain T-2 was inoculated into barley and lettuce plants, pathogenicity was not observed from germination to harvest, indicating that *Leptosphaeria* sp. strain T-2 is not pathogenic (Fig. 8). It is thought that a cascade of events may have facilitated the establishment of symbiotic relationships between plants and microorganisms. The fact that very few closely related species are known to exhibit these effects on the host is due to the evolutionary relationship between the loss of virulence and the acquisition of PGP effect in *Leptosphaeria* spp., which infects plants during the differentiation process of strain T-2. Hence, further research is required to compare strain T-2 with the pathogen *L. maculans* at the whole-genome level. As *Leptosphaeria* spp. are generally known as plant pathogens, the existence of PGP strains such as strain T-2 is a novel discovery. Switching between the pathogenic, neutral, and beneficial effects of endophytic fungi appears to occur frequently, and strain T-2 may also exhibit this kind of mechanism. As the relationships between endophytic fungi and plants are diverse and complex, the mechanisms underlying the growth-promoting effects of endophytic fungi are also diverse. This raises new interest in the mechanisms underlying the various effects of strain T-2 on plants.

However, environmental deterioration due to recent developments and climate change has become a serious problem that threatens sustainable agriculture, and rain-dependent (non-irrigated) agriculture is becoming difficult due to water scarcity in drylands that worsen annually. For this reason, dryland agriculture requires improved irrigation equipment; however, due to limited water resources, one approach is to make plants tolerant to water stress. Additionally, if irrigated agriculture continues, salt in the irrigation water may remain in the soil, and salt in the ground may accumulate on the ground surface owing to capillary action. Water potential is an important factor for plant growth, and the classification of soil moisture has been studied in detail from the perspective of water absorption and utilization by plants. The maximum water capacity is 0 MPa, the field water volume is − 0.006 MPa, the growth inhibition moisture point is − 0.1 MPa, and the initial withering point is − 0.6 MP (Ministry of Agriculture, Forestry and Fisheries n.d.). Therefore, at 20% PEG concentration (− 0.51 MPa), water stress was set around the initial wilting point in this study. The results showed that strain T-2 not only elicited growth-promoting effects but also conferred water and salt stress tolerance to its host plant (Figs. 5–7). In addition, the lettuce plants inoculated with strain T-2 grew under moisture conditions of − 0.1 MPa, which is the moisture point that inhibits plant growth. Interestingly, the barley from which the strain T-2 was isolated originated from a non-arid field in Japan, and the strain T-2 enhanced the plant's tolerance to environmental stress. This result may be attributed to the effects of strain T-2 on the biochemical properties of the plant. Furthermore, strain T-2 can confer tolerance to high salt concentrations up to approximately 4,000 mg/ kg; therefore, it is useful for agricultural production on salt-accumulating soils. Long-term salt stress inhibits plant growth, accelerates senescence, and causes death (Redman et al. 2002; Iqbal and Ashraf 2013). The common countermeasures to remove salt from the soil for salt-accumulating soil include physical methods, such as soil stripping to remove salt, and chemical methods, such as using soil conditioners. Phytoremediation is another biological method involving the absorption of salt by plants. Although some plants are highly salt tolerant, most crops that humans and animals use for nutrition are relatively sensitive to high salinity stres (Ondrasek et al. 2011). Therefore, developing effective methods to enhance the salt tolerance of plants is important for agricultural production. Therefore, the use of endophytic microorganisms as biofertilizers is expected to be an efficient means of sustainable agriculture in arid and highly saline areas (Radhakrishnan et al. 2014).

## Conclusion

Endophytic fungi live symbiotically with plants, promoting plant growth and improving plant health under stressful environmental conditions. This study reports the discovery of a newly isolated endophytic fungus, *Leptosphaeria* spp. strain T-2, which promoted plant growth and reduced host plant damage caused by drought and salt stress. Various effects are observed during the early stages of growth and persisted even during long-term cultivation. Therefore, strain T-2 can be considered an excellent endophytic fungus for use in dryland agriculture.

## Materials and methods

### Isolation of endophytic fungi

Endophytic fungi were isolated from the roots of barley grown in fields located in four prefectures in Japan (Table 3).

**Table 3:** Barley root collection site for endophytic fungus isolation

Prefecture, City	Day	Number of fields	Number of roots
Yamanashi, Hokuto	2018. 01.26	9	83
Ibaraki, Chikusei	2018.03.13	6	52
Gunma, Maebashi & Takasaki	2018.03.20	3	38
Toyama, Tonami	2018.04.04	4	38
Yamanashi, Hokuto	2018.05.15	2	16
Yamanashi, Kai	2018.05.30	1	5

These barley plants were grown in a field, dug up along with the surrounding soil, and the soil attached to the roots was carefully sifted and brought back to the lab. The barley roots were washed with tap water, and healthy, thick roots growing from the plant were selected and cut into 0.5-cm-long fragments. The root fragments were surface-sterilized through the following steps: first, the root fragments were vortexed with 70% ethanol for 1 min and then vortexed with 1% sodium hypochlorite solution for 1 min; afterwards, the fragments were washed with sterile water and then vortexed thrice. To isolate endophytic fungi, the root fragments were grown on antibiotics-supplemented potato dextrose medium (PDA) at 25 °C. The colonies that developed from the root fragments were transferred to a new medium and cultured for isolation.

### Screening of plant growth–promoting (PGP) fungi

The isolated endophytic fungi were co-cultivated with barley to assess their PGP effects. Barley seeds were surface-sterilized as follows: the seeds were stirred with a large amount of distilled water overnight and then stirred with 1% calcium hypochlorite solution for 20 min; subsequently, the seeds were washed with sterile water thrice. The seeds were infected with the isolated endophytic fungi by germinating them on the colonies of each fungal strain on PDA medium and exposing them to fungal cells for 2 days after germination. The surface-sterilized seeds germinated on 1% agar served as the control. Three germinated seeds were planted per pot containing 400 g of air-dried soil; the relative water content (RWC) in the pots was adjusted to 45%. The plants were cultivated in an artificial climate chamber with the following conditions: 25/15 °C (light/dark) temperature, 13/11 h (light/dark) photoperiod, 50% relative humidity, and 400 μmol/m^2^/s photosynthetic photon flux density. The plants were collected after the 28-day cultivation period, and the following parameters were measured: shoot length (SL), root length (RL), and fresh weight (FW). Subsequently, the dry weight (DW) of the plant samples was determined after drying them in an oven at 60 °C for 5 days. Based on these growth parameters, the barley samples that grew better than the control were selected. The fungal strains that exhibited growth-promoting effects on the barley plants were selected among the isolated endophytic fungi.

In an additional cultivation experiment, barley seeds were inoculated with four selected fungal strains (Y2-19, Y1-25, G-4, and T-2) and *Piriformospora indica*, which is known as a PGP fungus. A germinated barley seed was planted per pot. The barley plants were cultivated for 28 days under the same conditions as described previously. The plants were harvested after the cultivation period had ended, and the following growth parameters were measured: SL, RL, and FW and DW of the shoots, roots, and panicles.

### Plant growth under natural environmental conditions

The fungal strain T-2 that was grown for 10 days was hollowed out from its culture plate with a cork borer and cultured in 15 mL of potato dextrose broth per 6 sections at 25 °C for 7 days. Lettuce seeds were grown on Jiffy-7® (Sakata Seed Corporation, Tokyo, Japan) peat. A colony piece of strain T-2 was placed in the center of sterilized Jiffy-7® peat and cultured at 25 °C for 7 days. The lettuce seeds were surface-sterilized by shaking them with the following solutions, in this order: 70% ethanol at 90 times/min for 1 min, 1% sodium hypochlorite solution at 90 times/min for 20 min, and sterile water. Afterwards, the seeds were washed thrice. The experimental sterilized seeds were sown on strain T-2 colonies cultured on 1/10 Murashige–Skoog (MS) medium, while the control seeds were sown on 1/10MS medium. Germinated seeds were transplanted into Jiffy-7® peat and grown at 25 °C for 7 days. Lettuce roots were inoculated with strain T-2 by growing them in contact with strain T-2 colonies cultured on Jiffy-7® peat. The lettuce seedlings were planted in a pot containing 10 kg of field soil, watered daily, and cultivated under normal conditions for 5 weeks. The SL, FW, and DW (after drying in the oven at 80 °C for 7 days) of the harvested lettuce plants were measured.

### DNA extraction and sequence analysis

The isolated endophytic fungi were identified by performing PCR–restriction fragment length polymorphism (RFLP) analysis and sequence analysis. The isolated endophytic fungi were cultured on PDA medium, and DNA was extracted from their hyphae. Fungal cells were collected from the colony and transferred to a tube containing 200 µL of DNA extraction buffer. The fungal cells were disrupted with a homogenizer pestle, and the resulting suspension was incubated at 60 °C for 15 min. Subsequently, the suspension was chilled by placing it on ice for 20 s; then, 150 µL of CIA (chloroform-isoamyl alcohol; 24:1) was added to the suspension and mixed well using a vortex mixer. Afterwards, the suspension was centrifuged at 13,000 rpm and 25 °C for 15 min. The supernatant liquid was transferred to a new tube; the same amount of isopropanol was added, and the tube was allowed to stand at 4 °C for 20 min. Then, the tube was centrifuged at 13, 000 rpm and 4 °C for 15 min, and the liquid was discarded. Afterwards, the tube was washed rapidly with 300 µL of 70% ethanol that had been previously chilled at $-$ 20 °C; then, it was centrifuged at 13,000 rpm and 4 °C for 15 min, and the ethanol was discarded. The tubes were placed upside down, with open lids, to allow them to dry at room temperature for 30 min. Subsequently, 50 µL of 1/10 Tris–EDTA buffer was added to the tubes to dissolve the DNA precipitate. The internal transcribed spacer (ITS) region of the extracted DNA was subjected to PCR–RFLP analysis.

ITS1 (5′-TCCGTAGGTGAACCTGCGG-3′) and ITS4 (3′-TCCTCCGCTTATTGATATGCT-5′) were used as the PCR primers. The PCR mix consisted of the following: 9.5 µL sterile water, 12.5 µL GoTaq® Green Master Mix, 1.0 µL of the forward primer (ITS1), 1.0 µL of the reverse primer (ITS4), and 1.0 µL of the DNA sample.

The PCR settings were as follows: 95 °C (5 min), 94 °C (30 s), 55 °C (30 s), 72 °C (30 cycles, 1 min each), and 72 °C (10 min). The PCR products were subjected to RFLP analysis. Two types of restriction enzymes were used: *Alu* I and *Hinf* I. Two 1.5-mL tubes were prepared; 1.0 µL of buffer, 4.0 µL of sterile water, and 4.5 µL of the PCR product were added to each tube. Then, 0.5 µL each of *Alu* I or *Hinf* I was added to either of the two tubes. The tubes were incubated at 37 °C for 30 min. After the reaction, the mixture was cooled on ice, and 3 µL of 2 × loading buffer was added and mixed. Electrophoresis was performed at 100 V for 30 min on 2% agarose gel; the resulting bands were confirmed through GelRed® staining. The strains subjected to sequence analysis were selected based on the morphological characteristics of the cultured fungal strain colonies and the results of the PCR–RFLP analysis.

For sequencing analysis, extracted DNA of the selected strains was amplified by PCR, and PCR products were purified using the High Pure PCR Product Purification Kit (Roche Diagnostics, Penzberg, Germany), according to the manufacturer's protocol. Afterwards, 3.0 µL of the purified PCR product and 0.5 µL of 2 × loading buffer were mixed and electrophoresed on a 1.5% agarose gel at 100 V for 20 min. The resulting band was confirmed through GelRed® staining. The purified PCR products were sent to Macrogen Japan (Macrogen Japan Corp., Tokyo, Japan) for sequencing; the sequence results were identified using the basic local alignment search tool (BLAST) provided by National Center for Biotechnology Information.

### Phylogenetic analysis

The strain T-2 was sequenced not only in the ITS region but also in the SSU region for phylogenetic tree construction. The SSU region was also amplified by PCR. NS1 (5′-GTAGTCATATGCTTGTCTC -3′) and NA8 (3′-TCCGCAGGTTCACCTACGGA -5′) were used as the PCR primers. The PCR mix consisted of the following: 9.5 µL sterile water, 12.5 µL GoTaq® Green Master Mix, 1.0 µL of the forward primer (NS1; 5'-GTA GTC ATA TGC TTG TCT C-3'), 1.0 µL of the reverse primer (NS8; 5'-TCC GCA GGT TCA CCT ACG GA -3'), and 1.0 µL of the DNA sample. The PCR settings were as follows: 95 °C (4 min), 94 °C (1 min), 58 °C (1 min), 72 °C (35 cycles, 2 min each), and 72 °C (8 min). PCR products were electrophoresed on 1% agarose gel at 100 V for 20 min; the resulting bands were confirmed through GelRed® staining. The PCR products were sent to Macrogen Japan (Macrogen Japan Corp., Tokyo, Japan) for sequencing.

After subjecting the ITS and SSU nucleotide sequences to BLAST sequence homology estimation, MEGA11 was used to create a phylogenetic tree based on the maximum likelihood estimation of the nucleotide sequences obtained.

### Re-isolation of strain T-2

To confirm whether the inoculum of strain T-2 had infected the roots of the plants, the inoculated strain was re-isolated from the roots. The lettuce seedlings inoculated with strain T-2 using the method described in Section "Plant growth under natural environmental conditions" were grown in Jiffy-7® peat. After the cultivation period had ended, strain T-2 was collected from the surface-sterilized roots as described in Section "Plant growth under natural environmental conditions". The colonies that grew on PDA plates were collected. Genomic DNA of the obtained fungal strain was extracted using a DNA extraction kit (Fungal/Bacterial Microprep kit, Quick-DNA; Zymo Research, Irvine, CA, USA), according to the manufacturer’s protocol. Sequence analysis was performed by Macrogen Japan, and the obtained nucleotide sequences were subjected to BLAST analysis.

### Microscopic observation

Microscopic observations were conducted to confirm whether the hyphae of strain T-2 colonized the root tissue of barley. Barley seeds were surface-sterilized, germinated on strain T-2 colonies on PDA, and exposed to the strain T-2. Non-inoculated barley seeds were germinated on water–agar medium. To inoculate barley roots with strain T-2, the germinated seeds were grown on the colonies of strain T-2 for 7–14 days. The barley roots were cut into 1-cm-long fragments and dyed. The root fragments were immersed in 10% potassium hydroxide solution, sealed, and kept at 60 °C overnight. The roots fragments were washed with distilled water, immersed in 0.05% direct blue solution, sealed, and left at 60 °C for 6 h for staining. Subsequently, they were washed with distilled water to remove the excess staining solution. Slides were prepared from the stained root fragments and observed under a microscope.

### Drought and salinity stress tolerance

The effects of strain T-2 on host plants under drought and salinity stress were determined by performing plate assays and pot experiments. For drought stress, lettuce plants were grown in 1/10MS medium supplemented with polyethylene glycol (PEG8000) to adjust the water potential of the medium. Media supplemented with PEG8000, which is less toxic to plants, are also frequently used in tissue culture experiment (Weele et al. 2000). The water potential of the culture medium was determined as described by Michel (1983). Strain T-2 and lettuce were co-cultured in 1/10MS medium supplemented with PEG8000 (0%–20%). For salt stress, strain T-2 and lettuce were co-cultured in 1/10MS medium with NaCl (0–70 mM). Strain T-2 was cultured in the medium for 2 weeks, and surface-sterilized lettuce seeds were sown onto the strain T-2 colonies. After germination, the plants were grown in a climate chamber for 7 days for drought stress and 10 days for salinity stress. After cultivation, the SL, DL, FW, and DW (after drying at 60 °C for 7 days) of the lettuce seedlings were measured. Environmental stress tolerance tests of strain T-2 were performed using pot cultivation tests. Lettuce seedlings were planted in Jiffy-7® peat, as previously described. The germinated seeds, which were exposed to the strain T-2 for 2 days after germination, were sown to be in contact with strain T-2 in Jiffy-7® peat; afterwards, the seedlings were cultivated in an artificial climate room at 25 °C for 7 days.

For pot experiments, lettuce seedlings were planted in 400 g of air-dried soil. Soil RWC was adjusted from 55 to 15% for the drought stress tolerance test. To induce salinity stress, the salt concentration in the soil was adjusted to 0–100 mM/100 g of soil. For water management during plant cultivation, the weight of the pot was measured daily and distilled water was added to compensate for weight loss. The seedlings were cultivated in an artificial climate room at 25 °C for 28 days. After the cultivation period had ended, the SL, DL, FW, and DW (after drying at 60 °C for 7 days) of the harvested plants were measured.

### Statistical analysis

The data were subjected to statistical analysis by Student *t*-test (*P* < 0.05). Statistical analyses were performed with SPSS Statistics Version 16.0 (SPSS Inc, Chicago, USA).

## Data Availability

All data analyzed during this study are included in this published article and sequencing data deposited to DDBJ database. In addition, data will be made available on request.

## Abbreviations

PDA: Potato dextrose agar
RWC: Relative water content
RFLP: Restriction fragment length polymorphism
BLAST: Basic local alignment search tool
PEG: Polyethylene glycol
PGP: Plant growth–promoting
ITS: Internal transcribed spacer
